# Behaviour Change Techniques: An Application to Increase Employees’ Willingness to Accept a Salary Reduction

**DOI:** 10.3390/bs14100924

**Published:** 2024-10-10

**Authors:** Emma Goelema, Muhammad Umar Boodoo, Fadi Makki, Ahmad Baasiri, Jana Kontar, Georgi Kirilov, Ivo Vlaev

**Affiliations:** 1 Warwick Business School, University of Warwick, Scarman Road, Coventry CV4 7AL, UK; 2 Boston Consulting Group, Washington, DC 20005, USA; 3 Nudge Lebanon, GS Building, Sit Nasab Street, Beirut P.O. Box 113-5402, Lebanon; 4 Department of Social, Organizational, Clinical and Educational Psychology, Faculty of Philosophy, Sofia University, 1000 Sofia, Bulgaria

**Keywords:** pay reduction, behaviour change technique, mixed method, employer–employee relationship

## Abstract

During the COVID-19 pandemic, many organisations worldwide asked their employees to accept a temporary salary reduction to manage the financial consequences of the unprecedented event. In this paper, we use a CEO’s salary reduction announcement to all employees and investigate whether a behaviour change intervention using five selected Behaviour Change Techniques (BCTs) increases expat employees’ overall willingness to accept a temporary salary reduction. We use mixed qualitative and quantitative methods, including survey and experiment, to test our hypotheses and frame our results. The results show that, while the direction of impact was positive, respondents were overall not significantly more likely to accept a temporary salary reduction. However, a significant effect was found for the individual BCT ‘Modelling’. Participants were significantly more likely to accept a pay cut if they knew their senior management took a larger cut than asked of participants. The outcomes of this study suggest that the availability of a financial buffer and the strength of the employer–employee relationship play a, possibly more important, role in employees’ willingness to accept a pay reduction. In addition, leadership matters as this study shows that people are more willing to accept a salary reduction when senior management leads the way.

## 1. Introduction

The COVID-19 pandemic has not only caused an enormous health care crisis but has also resulted in an unprecedented economic impact on both organisations and employees worldwide. Extreme cost-cutting measures, which before the crisis were rare and often limited to a single organisation in severe financial distress, became common practice for entire industries struggling to contain the economic fallout of the pandemic. One such practice was the (temporary) reduction of employees’ salaries and allowances. In August 2020, almost one-fifth of senior UK managers indicated to have accepted a salary reduction during the pandemic [1]. Moreover, in December 2020, a total of 9.9 million jobs from 1.2 million employers in the UK were furloughed as part of the government’s job retention scheme and the total value of claims made reached £46.4 bn [2]. Under this scheme, employees took unpaid leave while the government covered up to 80% of their regular wage, creating a situation where employees effectively accepted a temporary 20% pay cut.

Such government support actions were not available in all parts of the world. As a Gulf Cooperation Council (GCC) country reliant on foreign workers, Qatar instructed its state-backed entities to reduce monthly salary costs for foreign workers by 30% through pay-cuts and lay-offs [3,4].

While a financial sacrifice from employees is often part of suggested cost reduction measures, little research has been conducted from a behavioural science point-of-view on what increases or reduces employees’ willingness to accept a (temporary) salary reduction. This study’s aim is to contribute to closing that gap by using the Behaviour Change Wheel (BCW) framework [5], a well-established tool within the behavioural sciences, to better understand the important considerations for (expat) employees. The stepped approach of the BCW allows us to first execute a general assessment of which COM-B components (capacity, opportunity, or motivation) influence employees’ willingness to accept a (temporary) salary reduction. This COM-B analysis will then be used to select the “correct” Behaviour Change Techniques (BCTs) to include in a planned experiment on expat employees’ willingness to accept a (temporary) reduction after reading an CEO’s message announcing the reduction. A BCT is seen as an ‘active ingredient’ of an intervention “designed to alter or redirect causal processes that regulate behaviour” [5].

The COM-B model and BCW have been praised by many for their contribution in providing a standardised approach to behavioural analysis and the design of an intervention [6,7]. Some authors [8,9] critique the one-size-fits-all approach of the BCW as it does not address the variability of individuals and, moreover, uses broad constructs that are difficult to falsify. Others point to the focus on the individual to establish behaviour change as a potential weakness as the context and community level matter as well [10,11]. Nevertheless, the COM-B model and BCW have been praised by many for their contribution in providing a standardised approach to behavioural analysis and the design of an intervention [6,7].

The focus of this study will be on a written CEO’s message announcing a temporary salary reduction and whether the inclusion of certain BCTs in this message can influence employees’ willingness to accept a salary reduction. Applying the BCW approach to the topic of salary reduction acceptance has not been done before and the outcomes could be relevant for both individual organisations as well as public policy makers. Moreover, the proposed target group and country of the study place the focus on a less studied group of employees: expats in Qatar.

Our literature review, presented in the next section, covers the existing knowledge on the factors that motivate employees to agree to salary reductions (e.g., the employer-employee relationship, perceived loss size, leadership). The novelty of our study lies in the use of a comprehensive and systematic framework—the Behaviour Change Wheel—which incorporates all possible factors (mechanisms) that could influence behaviour. This approach allows us to uncover additional factors and suggest tailored interventions (techniques) to address them, thereby promoting behaviour change. By applying this theory-driven framework, we aim to both identify new mechanisms of action and reaffirm known ones, while also proposing targeted interventions to facilitate the desired behaviour change.

## 2. Literature Review

### 2.1. The Employer–Employee Relationship

The ‘psychological contract’ that describes the “individual’s beliefs regarding the terms and conditions of a reciprocal exchange agreement between that focal person and another party” [12] has been used to understand the employer–employee relationship [13]. The individual’s belief that there is an ‘obligation of reciprocity’ [12] (an (in)tangible compensation for their efforts) lies at the core of the psychological contract, and any real or perceived divergence from the contract can cause a contract violation. Violations can come in many shapes and forms, but one of them is to change the compensation criteria during the contract period [14]. Halkos and Bousinakis’ study [15] on employee’ stress and dissatisfaction during Greece’s economic crisis also confirms this and identifies the acceptance of a salary reduction as one of the causes for employees’ stress. However, as it is the individual’s experience that determines whether the employees see the event as a breach of contract, Rousseau identifies three elements that affect whether a divergence from the contract will be seen as a violation: monitoring (employee/employer actually considers whether the event outcome is different from the expected outcome), relationship strength, and perceived size of loss [14]. The two latter factors will be discussed in more detail as they are specifically relevant for employees’ willingness to accept a salary reduction.

### 2.2. Relationship Strength

Rousseau proposes that when a strong relationship and trust exist between employer and employee, this gives both parties a higher tolerance for behaviour or events that could be seen as a breach of the psychological contract [14]. This could imply that employees are more willing to accept a pay reduction when they have had a strong relationship with the employer in the preceding period. There is some evidence that this is indeed the case. Osborne et al.’s [16] study looked at 953 faculty members from four different public universities in California who underwent an unpopular system-wide salary reduction. They found that employees who felt that their sub-group had been treated with respect were less likely to protest the pay cuts compared with employees who felt that their sub-group had been treated with a low level of respect by university administrators.

### 2.3. Perceived Size of Loss

Two aspects of the size of the perceived loss are important. Firstly, small discrepancies between expectation in actual outcome tend to be more accepted than larger discrepancies [14]. This explains the common observation that people prefer a year without bonus payment to a reduction of their base salary. Secondly, the perceived breach of contract is larger when the amount of harm done to the party increases. Therefore, a base salary cut, which will presumably affect financial planning more than a one-off reduction in variable pay, is likely to be seen as a severe breach of contract. This also suggests that willingness to accept a pay reduction is affected by the amount of perceived ‘harm done’. It could therefore be that employees with a larger perceived financial buffer are more willing to accept an income reduction. At the same time, Rynes et al. [17] warn that pay could often be more important to employees than some company employee surveys suggest. Their analysis of several existing studies showed that, when asked directly, employees would rank pay as a motivator fifth, whereas “an analysis of meta-analytic studies of actual behaviour in response to motivational initiatives nearly always shows pay to be the most effective motivator” [18]. The authors indicate that it is likely that socially desirable responding could account for this difference.

Related to the above is also the concept of ‘fairness’. Kahneman et al. introduced the concept of ‘dual entitlement’ which has a similar premise as the psychological contract: it “governs community standards of fairness: transactors have an entitlement to the terms of the reference transaction and firms are entitled to their reference profit” [19]. In the research, participants were asked by phone survey to judge hypothetical situations as fair, acceptable, or (very) unfair. Participants judged situations where an employee had to accept a lower wage because others earned less as unfair, while they judged a reduction in income fair when the “reference profit of a firm is threatened” [19]. Even though the research took place over 30 years ago, there is merit in the idea that a salary reduction will be deemed more acceptable in a situation where the firm itself is under pressure as well.

## 3. The Role of Leadership

An extension of the factors mentioned in the first segment is the role that leadership plays in the willingness to accept a reduction in salary. General leadership literature as well as some specific research on the acceptance of pay cuts point to three concepts relevant to this research: the leader as a role-model, the impact of empathy, and the importance of explaining the situation to employees.

Especially in the leadership literature focussing on culture change within a company, it is often recommended that leaders start to display the behaviour they want to see amongst employees themselves [20,21]. Similarly, articles advising leaders how to survive the financial impact of the pandemic recommend senior leadership to lead by example and to take a (larger) salary reduction themselves [22,23]. “If you don’t [take a pay cut], there is a danger that your staff will feel like saps, making sacrifices while the C-suite continues unaffected.” [22]. In this case, leaders setting the example is expected to increase prosocial behaviour among employees. This is also called ‘self-sacrificial leadership’ where leadership places the collective welfare above their personal interests [24]. De Cremer et al. found evidence that this type of leadership “motivates follower prosocial behaviour, particularly amongst followers with a prevention focus” [25]. The researchers distinguish between followers with a ‘promotion focus’ (people looking for positive personal outcomes) and followers with a ‘prevention focus’ (people focused on avoiding negative outcomes and who think more about social consequences as well) [25]. While the outcome of De Cremer et al.’s study [25] is interesting, it is important to realise that it did not use a financial sacrifice from the leader as an example of self-sacrificial leadership. Instead, it used cases where leaders either increased or decreased their time contribution to group work during company restructuring and the amount of personal risk leaders are willing to take for the benefit of the organisation. Note that one could argue that a financial sacrifice in these unprecedented times does not necessarily come from altruistic motives alone but also protects the leader’s public image and thus personal interest in the long term.

An interesting article that deals with some of the factors explained above and the effect of a salary reduction is Greenberg’s study [26] on employee theft in reaction to a temporary salary reduction. As it is one of the few studies actually looking into employee behaviour in relation to a salary reduction, this study will be described in a bit more detail. In his study, employees from three US factories belonging to the same parent company were used to research whether employee theft differed between plants after receiving a different type of salary reduction announcement. Plant A received an ‘adequate explanation’ and Plan B an ‘inadequate explanation’. Plant C was used as a control group as orders had stayed the same and no reduction was needed in that factory. The ‘adequate explanation’ consisted of a combination of several factors, which can be related to current BCTs, compounded in a meeting announcing the temporary salary reduction:

While pilfering increased in both plants, the study also indicated that a “critical moderator” of this tendency was found in providing an adequate explanation for the reduction. “The use of adequately reasoned explanations offered with interpersonal sensitivity tends to mitigate the negative effects associated with the information itself” [26]. The positive effect of the use of evidence on message effectiveness is also widely acknowledged in the broader academic literature [27,28,29].

Another element that possibly contributed to these results is the use of a different messenger (source effect), in this case the President or Vice-President. Wilson and Sherrell [18] found in their meta-analysis of several studies of source effects on persuasion that the extent to which persons perceive the source to have characteristics such as credibility, expertise, trustworthiness, physical attractiveness, and ideological similarity impacts his/her persuasive abilities.

## 4. Salary Reduction as Reverse Donation

Taking the perspective of seeing a salary reduction as a reverse donation opens up analysis of the topic to a wealth of literature on actions and BCTs to increase volunteer participation or donations. However, some would argue that the voluntary aspect as well as the ‘cause’ are significantly different. Two theoretical concepts that play a role in this are loss aversion [30] and the endowment effect [31]. Kahneman’s revolutionary prospect theory states that a reference point exists, in our case the salary before the reduction, and that “losses loom larger than gains” [30]. In contrast, during a donation, the money is used as a vehicle to establish an exchange: the positive feeling that comes with making a donation. The same warm feeling might not be experienced when asked to accept a salary reduction, a ‘donation’ of money you possibly would have liked to spend otherwise. This is why this section highlights below some of the most promising BCTs from the donation literature for our study, but their appropriateness to the topic will have to be further studied in the COM-B survey in Chapter 3 of this dissertation. Albeit relevant to donation research, the use of a (dynamic) social norm will not be discussed in further detail because in the context of a company-wide salary reduction, all employees are informed of a salary reduction at the same time with no prior reference to conform to.

### 4.1. Message Framing—Consequences

In their messaging, charities have a choice to highlight either the negative consequences of non-donation (loss frame) or the positive effects donation can have on the cause (gain frame). Academics differ on which of these two frames is most effective. Those who consider negative appeals to be more effective focus on the negative emotions that arise from reading such a message. It is suggested that feelings such as distress and guilt increase ‘helping behaviour’ [32]. Moreover, this ties in with the loss aversion concept where individuals consider the prevention of negative consequences more important than gaining from positive consequences to a similar extent. Those in favour of positive framing highlight the positive emotions donators will get (the ‘warm glow’) when they see the positive impact of their donation [32].

One interesting point raised in this debate is that effectiveness may also depend on what exactly is being measured. Erlanddson et al. [32], discovered that some studies focus on the attitude (favourable or not) towards the cause and charitable organisation, while other studies measured actual donating behaviour or self-rated helping intentions. They suggest that a negative framing is more likely to increase actual donation behaviour, especially when participants do not expect to establish a relationship with the charity.

Another possible contributing factor to the differing effectiveness of one or the other message type is the relation between the topic of the message and the “processing motivation and capacity on the part of the receiver” [33]. For example, Block and Keller demonstrated that “for in-depth processing, negative frames are more persuasive than positive frames” [34]. This could imply that a negative framing would be more effective in salary reduction announcements as the processing motivation would be high and the opportunity to process unconstrained. Das et al. [33] looked deeper into this topic and found that “the relevance of the present charity was perceived to be higher when the message combined abstract, statistical evidence with a negative frame, or anecdotal, vivid evidence with a positive frame” [25]. A limiting aspect of this study, however, is the fact that researchers used (higher-educated) college students for their study who are possibly more used to interpreting statistical information.

### 4.2. Goal Attainment and Optimism

Similar to donating, accepting a salary reduction can be seen as a social dilemma: a situation where the immediate personal interest is at odds with long-term collective interests [35]. The degree to which one feels that the individual contribution helps to reach the collective goals (perceived efficacy) and how much value the problem has for the individual (whether it is involved or impacted) are important aspects of increasing cooperation in such situations [36]. Das et al. [33] found evidence that donation intentions can be increased when an explicit reference to the “likelihood of goal attainment” is included in the persuasive message [25].

Related to this is the influence of optimism. Some schools of thought highlight the positive aspects of optimism such as resilience and prosocial behaviour [37,38,39] while others highlight the risk of optimism causing people to set themselves up for failure by overestimating their capabilities and believing in a good outcome [40,41]. Bortolotti [42] suggests a third, alternative perspective by suggesting that (unrealistic) optimism is useful when it motivates us to pursue a goal and enhances resilience (and thus willingness to accept setbacks).

## 5. Data and Method: COM-B Analysis

The literature review highlighted that little research has been conducted on employees’ willingness to accept a pay reduction and, of the ones available, much of the research was conducted in Western-oriented countries. Moreover, as the BCW approach has so far not been applied to this topic, it was decided to follow all relevant BCW steps. An important element in the process is the COM-B model analysis. Michie et al. propose that changing individual behaviour “involves changing one or more of the following: capability, opportunity, and motivation relating either to the behaviour itself or behaviours that compete with or support it” [5]. In order to gain a full picture of the research topic, both the employer’s and employee’s perspectives were investigated. Four interviews took place based on the COM components to capture the employer’s perspective. These then served as input, together with the literature review and the suggested COM-B Self-Evaluation Questionnaire [5], to develop the survey that was sent out to employees. All the elements combined then informed the overall COM-B analysis.

Study 1 is exploratory in nature, rather than hypothesis-testing. We applied a comprehensive and systematic framework—the Behaviour Change Wheel—which considers all potential factors (mechanisms) influencing behaviour. This approach allowed us to identify additional factors and propose interventions (techniques) to modify these factors, whether they act as barriers or enablers of behaviour change. The novelty of our approach lies in utilizing a comprehensive theory of behaviour change to both uncover new mechanisms of action and reaffirm known ones, while suggesting targeted interventions to promote the desired behaviour change. Study 2 is a hypothesis-testing study.

### 5.1. The Employer’s Perspective

In order to gain a better insight into the employer’s perspective on (expat) employees’ willingness to accept a (temporary) salary reduction, four individuals based in the GCC were interviewed: a CEO of a large conglomerate, an entrepreneur in the hospitality sector, an HR manager for a large oil and gas company, and a managing partner of a consulting company in the area of education.

Two indicated that their organisations had reduced base salaries recently (the other two reported that their organisations have not reduced base salaries so far). All had taken actions such as a hiring freeze, no bonus payment, or a reduction in allowances to assist in weathering the financial impact of the pandemic. The CEO specifically mentioned trying to keep their expatriate staff within the company, as he did not think it was fair to release employees during the pandemic. The entrepreneur indicated that she had had to defer payments and put people on part-time contracts, as well as change the job content of certain roles. She added: “We tried not to lose anyone. You work so hard to get people on board and the last thing you want is to lose them for something you never planned for”.

When asked what would be important factors, in their opinion, when looking at employees’ willingness to accept a salary reduction, two elements stood out. Firstly, each of them mentioned that they felt the company would have to present a clear case for the salary reduction explaining why a cut was necessary and explain (give evidence) that all other cost-cutting options had been exhausted. One remarked: “You would need to make very clear the reason why a reduction needs to take place. Employees will need to feel this is a valid reason. If they don’t feel it is valid, or they don’t understand and feel the problem, they will not relate to the proposed solution”. Secondly, all highlighted the importance of senior management sharing the pain and being a role-model. In addition, several mentioned that they would prefer an approach where the largest burden would be on the higher income levels and lower-level salaries would remain unaffected.

As for the importance of a good relationship between employer and employee, sentiments were mixed. Three emphasised it as being essential and gave examples of employees within the organisation being quite understanding and willing to accept a form of reduction in income. The other explained that in her pre-COVID experience, employees tend to say they value the relationship but that they will leave when a reduction is announced and other, better paid, opportunities are available in the market. She added that it could be that this might be different now that the pandemic “has made reduction in salary the norm” and reduced external options, thus changing the opportunity cost of leaving.

### 5.2. The Employee’s Perspective—Study 1: COM-B Survey among Employees

#### 5.2.1. Methods—Study 1: COM-B Survey among Employees

Participants: Due to time considerations, the exploratory phase of the research, and the nature of the topic, the survey was open to anyone. In total, 216 persons started the survey, but after removing incomplete entries, the final sample consisted of 155 participants, of whom 84 were expats (for whom the country of nationality was not equal to the country of residence). In the study, 91 males and 64 females participated; a similar male–female split was observed when the data were filtered to include only expats. More than 90% of participants were between 25 and 54, with over 50% being between 35 and 44 years of age (88 participants).

Around 90% of the participants were in paid employment and over 75% of participants indicated that they lived (very) comfortably on their present income. Over 60% had a European nationality, mainly the Netherlands and the United Kingdom. Europe and the Middle East were the most common locations of residency. Additional demographic details of the sample can be found in Appendix C.

Procedure and material: Participants for the online survey (in English) were recruited via different social media platforms (LinkedIn, Facebook) and individual recruitment by the researcher. The survey comprised three blocks:

1. Informed consent and demographic questions.
2. Questions on factors related to the different COM-B components influencing willingness to accept a salary reduction, adapted from the COM-B Self-Evaluation Questionnaire [5]. All survey questions are available in Appendix D.
3. A final open question asked participants to explain why certain factors were most important to them.

Respondents were asked to judge the importance of COM-B factors to them on a five-point Likert scale (1 = extremely important, 5 = not at all important). The component ‘physical capability’ was not addressed in the survey, as there is no physical barrier to accepting a salary reduction. Next to that, five statements on the domain ‘emotion’ from the Theories Domain Framework (TDF) [43] and on the importance of trust between employee and employer were added, as the review of the literature had pointed to these aspects playing a role as well, and it was felt that the COM-B questionnaire did not address these aspects sufficiently. The TDF provides a further level of detail, with each domain linking to a COM-B component. Participants were asked to identify on a five-point Likert scale to what extent they agreed with these statements (1 = strongly agree, 5 = strongly disagree).

Originally, Michie et al.’s self-questionnaire [5] only asked participants to indicate whether a question was important to them or not. This survey used a five-point Likert scale to better understand the level of importance of an item compared with other items. The use of a Likert scale has the advantage that it can be pre-coded and enhances the equivalence of answers [44]. In this case, a five-point scale was used to allow for sufficient options in answering while not overcomplicating the answering process for the respondent.

#### 5.2.2. Results—Study 1: COM-B Survey among Employees

Table 1 shows the results of the survey ranked relative to the mean score given to the item by all participants. Results of the ‘expat only’ group are given underneath the general score.

All factors except one received a ranking between 3 and 1 indicating that all statements were considered to be moderately to extremely important on average. This did not differ for the expat-only group. Similarly, the overall ranking of statements, but especially the top 7 items, were almost identical when comparing the expat-only group with outcomes related to all respondents.

Participants gave most importance to the idea of senior management having to share the pain as well. Not only did this statement come out at the top of the table but the variance is relatively small indicating that all participants shared a common view on this. The need to understand the reasons for the decision was a very close second and 149 participants ranked this statement as very to extremely important.

## 6. Discussion—Study 1: COM-B Survey among Employees

As the survey result suggests, salary reduction acceptance is a multi-faceted issue and all COM-B components potentially play an important role in increasing its acceptance. Quite striking is that, albeit by a slim margin, participants deemed senior management partaking in the reduction more important than even the explanation about why a reduction is required. This supports the notion in the literature that leadership being a role-model is essential in changing employees’ behaviour.

Another interesting outcome of the survey is that the expat ranking did not differ substantially from the overall results. This could imply that being an expat or not does not make a difference in individual’s willingness to accept a reduction or that other variables such as income level and nationality (also in regard to the support systems available in the country) play a larger role. In this study, the majority of participants had a European background and were generally living (very) comfortably on their present income.

Probably indicative of the context in which this survey was asked was participants’ responses to the need to be aware of an external event causing damage to the economy to increase acceptance. The pandemic and consequential economic impact has increased the salience of the topic of salary reductions and brought it into the spotlight. Interestingly, while the pandemic has hit entire sectors, participants find it important that other employees within the same company accept a reduction but do not give the same importance to the statement that people externally should undergo the same.

As for the consequences of the reduction influencing willingness to accept, respondents rated the awareness of possible job losses as less important compared with some other factors. Nevertheless, the need to be able to oversee the personal consequences came out in the top five. While the question was intended to be more focused on the financial effects of a reduction, participants may have interpreted it as also including the knowledge of whether they themselves would be at risk of unemployment. This could explain the relatively low ranking of the impact of fear of job loss by participants.

A last and unexpected outcome of the survey was the fact that participants still ranked the statement “I would have to know more about how to accept a salary reduction” in 13th place. This statement, intended to test psychological capability, was included as it formed part of the standard questionnaire. However, it was forecast that participants would indicate this as not at all important to them as it relates to the aspects such as signing a document or other relatively simple acts from the employee to approve and process a salary reduction. We suspect, however, that some respondents interpreted the question more in a ‘how to absorb the reduction in my daily life’ manner and therefore weighted it as (very) important.

## 7. Limitations—Study 1: COM-B Survey among Employees

This study was always intended to be a preparatory study and to be used as an input document for selecting the most promising BCTs for the final experiment. A conscious choice was therefore made to include a diverse range of individuals consisting of employed, unemployed, expats, and ‘locals’. This enabled the collection of data in a short amount of time but also affected its generalisability. The sample is not representative of Qatar’s expat population and skewed towards individuals with a European background and a sufficient to high level of income. Moreover, the recruitment of participants via social media and individual recruitment brings with it the issues of self-selection bias and non-response [44].

However, the value of this study lies in the novel approach taken to study the topic of willingness to accept a salary reduction. The systematic review of COM-B components on this topic has not been done before and gives interesting insights by bringing a multitude of factors together that have before only been analysed separately in the academic literature, giving a more all-round perspective on the topic. Moreover, Ref. [5] stress the importance of trying to obtain the best well-rounded picture possible within the prevailing time and resource constraints before selecting specific change techniques.

## 8. COM-B Analysis: Identifying What Needs to Change

Incorporating the literature review, employer interviews, and the employees’ self-questionnaire study in the COM-B analysis leads to Table 2. As shown, all COM-B components, except for physical capability, are potential levers for change when it comes to employees’ willingness to accept a (temporary) salary reduction.

## 9. Identifying Intervention Options and BCTs

### 9.1. Intervention Functions

As the COM-B research indicated that all but one of the tested components could affect target behaviour, the next step in the BCW process, selecting the intervention options, was important to provide focus. Table 3 considers the applicability of the intervention functions based both on the APEASE criteria as well as the pre-defined mode of delivery (a CEO’s email message). Only intervention functions identified as appropriate according to both aspects were selected. These were Education, Persuasion, Incentivisation, Modelling, and Enablement. Definitions of the different intervention functions can be found in Appendix B.

### 9.2. Identifying Behaviour Change Techniques

The final step in the BCW approach was to select the BCTs best suited to the identified intervention functions. For this purpose, Michie et al. [5] have created an internationally recognised overview including 93 defined BCTs (BCT Taxonomy v1) [45] and the BCW approach provides a useful table where intervention functions and individual BCTs are linked together. A total of 21 BCTs linked to the identified intervention options were considered for this research and filtered through based on the APEASE criteria and suitability for the mode of delivery (detailed with definitions in Appendix D). Only those who passed both criteria were considered. Table 4 indicates which BCTs fulfilled both criteria.

When these outcomes were compared with information from the COM-B exploration phase, and specifically, the survey, it was observed that these fitted well with some of the high-ranking elements in the survey such as the importance to employees of senior leadership ‘Modelling’ the behaviour. At the same time, the BCTs identified did not clearly address two other factors that seemed to be of influence according to the literature and empirical COM-B research: the need among employees for a clear explanation of the reason for the reduction and the idea of a reduction causing anxiety among the target group.

### 9.3. Providing an Explanation

The first aspect, clearly making the case why a pay reduction is needed, does relate to the ‘Education’ intervention option and the BCT of providing information about the consequences. However, in this instance, it was not so much about consequences in the future but more about the reason why the behaviour is necessary. The ‘Knowledge’ domain within the more elaborate TDF framework [43] does cover this aspect more. Therefore, it was decided to replace the ‘social and environmental consequences’ BCT with the BCT ‘providing information about the reason behind the decision (explanation)’. Similar to Greenberg’s experiment [26], our experiment would test whether employees would be more likely to accept the reduction when a more elaborate reason ‘why’ was given.

### 9.4. Anxiety

Anxiety was another area that needed to be addressed but an exactly fitting technique from the BCT taxonomy could not be found. The BCT ‘Social Support (Emotional)’ had elements of the emotional support required but was not applicable to our mode of delivery, a written message to a larger group. Some authors, such as Hilton and Johnston [9], also mention that aspects such as empathy, warmth, and positivity have not been fully researched in a BCT context. Authors such as Marks et al. [46] contribute to this discussion by emphasising that behaviour change is not only about the ‘what’ but also by ‘who’, the behaviour change agent and its characteristics. “Hiding empathy within a BCT called ‘social support (emotional)’, as suggested by Michie et al. [45] is inaccurate. Empathy and the ability to strengthen the therapeutic alliance are necessary characteristics of a successful behaviour change agent” [46]. While Marks references a medical-oriented therapeutic alliance, similar importance is given to emphatic leadership and messaging in the leadership literature. Therefore, we added a fifth ‘BCT’ to our experiment: showing remorse for having to take the decision (empathy).

Table 5 summarises the five BCTs selected for inclusion in the experiment, including their definition and intended use in the experiment.

## 10. Study 2: A Behaviour Change Intervention to Increase Expat Employees’ Willingness to Accept a (Temporary) Salary Reduction

The main aim of this experiment was to assess the effectiveness of a message including the five selected BCTs in comparison with a message where these BCTs were not present. Consequently, this created a compound intervention involving multiple BCTs and, therefore, as recommended by the Medical Research Council’s guidance [47], the overall effect of the treatment had to be assessed too. For this reason, this study includes a primary hypothesis to look at the impact on overall willingness to accept a reduction in pay, as well as five secondary hypotheses relating to the individual BCTs.

Primary Hypothesis:

**H1.**  *The use of multiple Behaviour Change Techniques in a CEO’s email announcement significantly increases expat employees’ overall willingness to accept a salary reduction.*

Secondary Hypotheses:

**H2.**  *The use of Behaviour Change Technique—Knowledge/Explanation—in a CEO’s email announcement significantly increases expat employees’ willingness to accept a salary reduction.*

**H3.**  *The use of Behaviour Change Technique—Modelling (demonstrate behaviour)—in a CEO’s email announcement significantly increases expat employees’ willingness to accept a salary reduction.*

**H4.**  *The use of Behaviour Change Technique—Information about consequences (framing negative)—in a CEO’s email announcement significantly increases expat employees’ willingness to accept a salary reduction.*

**H5.**  *The use of Behaviour Change Technique—Empathy—in a CEO’s email announcement significantly increases expat employees’ willingness to accept a salary reduction.*

**H6.**  *The use of Behaviour Change Technique—Social Support—in a CEO’s email announcement significantly increases expat employees’ willingness to accept a salary reduction.*

In addition, the experiment explored for heterogeneity in effect size based on income levels, age, gender, and region of provenance. These exploratory analyses were conducted for both the overall willingness to accept a salary reduction and willingness to accept a salary reduction based on each individual BCT. However, due to the smaller sample size and the fact that income level was the main stratification variable, only the results relating to the income level of participants will be discussed further.

### 10.1. Methods—Study 2: A Behaviour Change Intervention to Increase Expat Employees’ Willingness to Accept a (Temporary) Salary Reduction

#### Participants

In total, 651 Persons opened the online survey. After removing incompletes (below 97% progress) and one failed attention check, a total of 140 expats employees in Qatar participated in this online experiment (103 male, 37 female). The majority of respondents were between 25 and 54 years old which fits well with the age distribution of expat employees active in Qatar. Over half of the participants were from the Asia/Pacific region and more than 80% indicated they had one or more adults dependent on their income (spouse, parents, adult child, or other family member who does not work). In total, 57% had one or more children under the age of 18. Participants’ levels of income were quite evenly distributed with 32 or 33 persons in each of the Comfortable, Getting By, Difficult, and Very Difficult groups; only the ‘Very Comfortable’ income bracket had substantially fewer respondents (10).

### 10.2. Procedure and Material

Participants were recruited via a Facebook advert (Appendix E), posting a link to the advert on some Facebook group pages and personal recruitment. In the advert, a link was provided to the online survey in Qualtrics. While this method can affect the representativeness of a study, it did provide a cost-effective way to reach the target group.

The survey consisted of four elements:

1. Informed consent (an explanatory PDF and informed consent questions that needed to be answered, otherwise the survey would not continue)
2. Demographic questions and an attention check question
3. The experiment testing the five BCTs and overall willingness to accept
4. A final question asking the participant to explain why the exact overall score was given

Table 6 shows the descriptive statistics. As seen, differences between the treatment and control groups are statistically insignificant.

In the experiment, participants were asked to imagine their current organisation had gotten into economically difficult times and to read five short parts of an e-mail message from their CEO announcing a 20% salary reduction for at least six months, until there are clear signs of recovery within the sector they work in. In each part of the message for the treatment group, a BCT sentence was added (full text in Appendix F). After reading a single part, they were asked to indicate how that part of the text had influenced their willingness to accept a salary reduction. A 7-point Likert scale was used in the experiment for this question (from 1, strongly decreased, to 7, strongly increased). It is generally recommended to use an uneven scale so that participants can choose a neutral point as well and, moreover, a scale between five and nine options seems to satisfy the need for a wide enough range for a nuanced answer while not overcomplicating the survey for participants [48]. For example, the BCT ‘Modelling the Behaviour’ was tested by adding the underlined text to the following paragraph in the treat group, and using the paragraph without the underlined for the control group:

*“The current conditions and organisational review have forced me to make a difficult but necessary decision. Starting next month, salaries will be reduced by 20% for at least six months until there are clear signs of recovery. Bonuses will not be paid over the current year and budgets for courses will be withdrawn until further notice. Health insurance coverage and allowances such as those for housing will remain unchanged. We believe senior management should lead by example and shoulder their part of the burden. Therefore, the salaries for the executive team and myself will be reduced by 30%, and we will also forego performance bonuses.”*

After having read the five text parts in isolation, participants were asked to read the complete message and rate on a scale from 1 (very unlikely) to 10 (very likely) how likely it was that they would accept a temporary salary reduction.

### 10.3. Randomisation and Statistical Analysis

The study had two between-subject conditions, the control survey and the treatment survey, including BCTs, which were randomly assigned to participants. Next to this, to reduce the risk that the order of the questions influenced participants’ ratings and thus overall results [49], options in Qualtrics were used to ensure the individual paragraphs (each containing a separate BCT) of the CEO message were presented in random order.

As the study was also interested in exploring heterogenous effects across income levels, it was necessary to achieve a balance in income levels across the control and treatment groups. Therefore, a sequential covariate non-adaptive randomization design, a type of block randomization, was used in Qualtrics [50].

The main dependent variable was the overall willingness of participants to accept a salary reduction, indicated by a coefficient treatment, which was measured on a scale from 1 to 10. The second dependent variable was how the willingness of participants to accept a salary reduction had been influenced by a particular behavioural change technique indicated by a coefficient treatment, which was measured on a scale from 1 to 7.

The results analysis was performed using multivariate linear regression, where control variables were added gradually to test the robustness of results to varying model specifications. The covariates were age, gender, and income level. *p*-values were computed based on the t-distribution. The Holm–Bonferroni method was applied to adjust for multiple testing. A *p*-value of 0.05 was used as the significance boundary to either accept or reject hypotheses.

The absence of multi-collinearity was assessed using the Variance Inflation Factor (VIF) with a threshold of VIF = 10. All regressions were estimated using Huber–White standard errors to ensure homoscedasticity.

### 10.4. Results—Study 2: A Behaviour Change Intervention to Increase Expat Employees’ Willingness to Accept a (Temporary) Salary Reduction

The results are presented in three main sections. The first investigates the overall willingness to accept a salary reduction and the second considers the impact of the individual BCTs on the same. The last section discusses the exploratory hypotheses related to income level.

### 10.5. Overall Willingness to Accept a Salary Reduction

Looking at the mean scores in Table 7, the overall willingness to accept a reduction for both the control and treatment groups was low with scores just over 4, rated on a scale of 1 to 10 (control 4.06 and treatment 4.39).

A multivariate linear regression analysis was performed to assess the impact of the intervention on overall willingness (Table 8), where overall willingness to accept a reduction was used as a dependent variable. After controlling for the financial situation of participants, the estimate of the impact of the intervention was 0.63 (SE = 0.47, *p* = 0.19), which was further reduced to 0.47 (SE = 0.49, *p* = 0.34) after also controlling for sex and age. While the direction of impact was positive, no statistical significance was observed. Thus, no support was found for H1, that the inclusion of specific BCTs in an CEO’s e-mail message leads to greater overall willingness to accept a salary reduction.

The participants’ financial situation correlates strongly with their willingness to accept. Compared with participants who described their financial situation as Very Comfortable, respondents in the Difficult or Very Difficult financial situation category were significantly less willing to accept a (temporary) salary reduction (b = −2.35, SE = 0.61, *p* < 0.01 and b = −2.43, SE = 0.7, *p* < 0.01).

### 10.6. Impact of the Specific BCTs

Multivariate linear regression analysis was also performed to determine the impact of each individual BCT. The outcomes of the analysis is given in Table 9 for both the results ‘without controls’ (only controlled for the stratification variable (financial situation)) and ‘with controls’ (including gender and age) and are discussed below.

The use of BCT1 ‘Information/Explanation’ did not improve willingness to accept a reduction (b = −0.065, SE = 0.25 *p* = 0.79). For this reason, H2 was not supported.

Including the BCT2 ‘Modelling’ in the text for the intervention group significantly increased their willingness to accept a (temporary) salary reduction (b = 0.75, SE = 29, *p* = 0.01). This significance was retained after adding the additional controls for age and gender (b = 0.68, SE = 0.30, *p* = 0.025) and consequently H3 was endorsed.

In the first instance, BCT3 ‘Negative Consequences’ showed some significance (*p* < 0.1); however, after controlling for additional controls, this significance was lost (b = 0.39, SE = 0.28, *p* = 0.17). This result led to the rejection of H4.

The application of BCT4 ‘Empathy’ in the e-mail message for the treatment group did not significantly increase their willingness to accept a temporary pay cut (b = 0.41, SE = 0.25, *p* = 0.107). Therefore, no support was found for H5.

The BCT ‘Social Support’ was examined in hypothesis six. No significant difference between the control and treatment groups was detected (b = 0.49, SE = 0.26, *p* = 0.065 and b = 0.5, SE = 0.27, *p* = 0.064); consequently, H6 was not sustained.

### 10.7. Exploratory Hypotheses: Influence of Participants’ Financial Situation on Overall Willingness and BCT Effectiveness

Heterogeneity analysis (Table 10) indicates that financial situation can also influence the impact of the intervention itself. Those who identified as ‘Getting By’ financially were significantly more willing to accept if receiving the message including the BCTs (b = 2.75, *p* < 0.01). Such an effect was not found for the other financial categories. A similar trend can be observed in the heterogeneity analysis for the individual BCTs (Table 10). Unadjusted *p*-values for four out of the five BCTs were statistically significant for the ‘Getting By’ category; however, none of the results remained statistically significant after adding further tests (Holm–Bonferroni).

Another noticeable result, given the fact that the financial situation is used as the stratification variable, is that the data suggest that BCT2 ‘Modelling’ had a significant impact not only on those in the ‘Getting By’ category but also on the ‘Very Comfortable’, while such an effect was not found for those participants who described their financial situation as ‘Difficult’ and ‘Very Difficult’.

## 11. Written Feedback from Participants

Similar comments to the themes of the COM-B survey came up when asking participants to explain why they gave the specific rating for overall willingness. Out of the 140 respondents, a total of 93 answered this question (treatment = 41, control = 51).

A frequently mentioned reason for low willingness to accept by both the control as well as the treatment cohort was the financial situation not allowing them to accept a reduction (n = 13): “with a already such a less salary, one can’t imagine surviving with a cut”. Within this group, some specifically mentioned the ‘end of service policy’, severance pay based on most recent income, and their worry that a reduction would only be a way for senior management to save on severance pay. Note that this reason was only given by those who had selected the ‘Getting By’, ‘Difficult’, or ‘Very Difficult’ description of their financial situation. In the ‘Comfortable’ and ‘Very Comfortable’ groups, it was mentioned by both the control as well as the treatment cohort that they felt the organisation should seek other ways of cost cutting before lowering salaries and that governmental support packages should make a salary reduction unnecessary (n = 11).

Comments related to the relationship with the employer covered both sides of the argument: some highlighted the willingness to accept a reduction if the employer had been trustworthy and kind previously (n = 2), while others emphasised the element of ‘sacrifice’ from the employee while working for the organisation and that this was a situation for the employer to show their appreciation of employees by not cutting salaries (n = 2). Others based their unwillingness to accept a salary reduction on their view that the employer had not been willing to share the financially good times (n = 2) with the employees before.

### 11.1. Discussion—Study 2: A Behaviour Change Intervention to Increase Expat Employees’ Willingness to Accept a (Temporary) Salary Reduction

This study’s main aim was to better understand whether the use of certain BCTs in a CEO’s message would increase expat employees’ willingness to accept a salary reduction. While the direction of impact was positive, an analysis of the results did not allow us to establish a statistically significant difference between those reading the control or treatment message. Several factors may have contributed towards this result.

Firstly, the current COVID-19 pandemic, which at the time of the experiment was in its eighth and ninth month since the first lockdown in Qatar, has brought concepts such as salary reductions, furloughing, and job losses to the forefront of working life. Where previously individual companies would occasionally get into financial difficulties, now complete sectors have been impacted. The salience of the pandemic and its consequences may have been so strong among the participants that the gist of both messages felt similar and was what they would expect for such an announcement. After all, the control message was still neutral and not harshly worded or unfriendly.

Secondly, as we asked participants to imagine that the message came from their own organisation, and the COM-B survey indicated that the emotional and financial relationship between employer and employee prior to a salary reduction was of great importance, it is possible that including BCTs at this stage to significantly improve willingness was ‘too late’ in the process and interventions to maintain and improve the relationship needed to have taken place at an earlier stage.

Lastly, it is likely that the concept of loss aversion played a role in respondents’ considerations. BCTs are often applied to situations where a clear benefit is to be obtained. As an example, losing weight can be very challenging, but the idea of weight loss is a clear positive benefit, and individuals are gaining something from their actions. In the case of accepting a salary reduction, something that was previously theirs is being ‘taken away’ for the benefit of keeping employment. However, in the current state, they have both employment and a higher salary. This could also explain why the mean overall willingness score is around 4 out of 10 (somewhat unwilling) for both groups despite the salience of the topic due to the pandemic. In this context, it raises the question whether a different wording of the question, e.g., ‘preparedness’ instead of ‘willingness’, would have given a different result.

For many behaviour-change interventions, both in experiments as well as in real life, it is the compound nature of several BCTs that makes the intervention successful. This is why this experiment’s primary hypothesis looked at overall willingness to accept the effects of the complete message with all five BCTs. However, on a secondary level, it is valuable to assess whether one specific BCT has a greater effect compared with the others. In this experiment, it was BCT2 ‘Modelling’ that stood out, being statistically significant at the 5% level while the others were not. This implies that the actions of senior leadership matter in this context, as the COM-B survey suggested as well. This aligns with the leadership literature that role modelling is important both from the perspective of social comparison increasing motivation and from the perspective of fairness where everybody is sharing the pain.

The fact that BCT1 ‘Information/Explanation’ did not seem to make an impact (in fact, the result had a negative direction in our experiment) is notable as Greenberg’s [26] experiment suggests that this BCT can be of great influence. One possible explanation is the aforementioned salience of the pandemic and its financial impact on organisation. It is quite possible that the control group did not need the extra sentence and had already assumed, reading between the lines, that the organisation was severely impacted by the pandemic and thus had a valid argument for reducing salaries. Moreover, as only limited additional information (one extra sentence) was given to the treatment group, it could be that actually more information was needed to have a significant impact.

As for the other three remaining BCTs (‘Negative Consequences’, ‘Empathy’, and ‘Social Support’), while our research did not prove that these significantly increased participants’ willingness to accept a reduction, they did all have a general positive direction of impact. It may therefore still be useful to include them in such a message.

An unexpected but very interesting result from the exploratory analysis was the influence of the participant’s financial situation on their willingness to accept a salary reduction. Those in the (very) difficult income level bracket indicated in their verbal feedback that they did not feel they had sufficient income to be able to absorb a reduction. This can explain the significantly lower overall willingness of both the control and treatment cohort to accept a reduction compared with the (very) comfortable groups. Furthermore, the end of service policy combined with a level of mistrust towards management could also have contributed to their low willingness. These aspects again reflect the findings of the COM-B survey, which highlighted the importance of having a financial buffer, either in income level or savings, and a good relationship with the employer. Moreover, this finding also highlights a topic not included in this experiment, namely that of domestic labour laws affecting people’s attitudes towards a pay reduction.

As for the impact of the intervention, we were surprised that although the general outcome was not statistically significant, there was one subgroup, namely ‘Getting By’, highly influenced by the experiment. Further analysis of the comments given by this group could not give any further clarity as to why this was the case. In addition, a closer look at the different regions represented in the control and treatment cohort for ‘Getting By’ showed that they were fairly similar in composition and thus cannot immediately explain the significant findings (Appendix G).

Another unexpected finding was the apparent split in the effect of BCT2 ‘Modelling’ between those in a better financial situation (‘Getting By’ and ‘Very Comfortable’) as opposed to those struggling to make ends meet (‘Difficult’ and ‘Very Difficult’ categories). It is possible that the results of the latter group can be explained through their lower overall willingness to accept a reduction. However, the reason why those in the ‘Very Comfortable’ category are significantly more likely to be positively influenced by BCT2 cannot be directly established from this study.

### 11.2. Limitations—Study 2: A Behaviour Change Intervention to Increase Expat Employees’ Willingness to Accept a (Temporary) Salary Reduction

While the use of a Facebook advert gave cost-effective access to a wide range of expat employees in Qatar, it is important to highlight how this method does affect the generalisability of the outcomes due to sample bias. Firstly, reaching out to participants in this manner is likely to lead to ‘self-selection bias’. An advantage of self-selection is that participants are likely to be more willing to fill out the survey; however, a downside of self-selection is that there is a possibility of overemphasis on a particular finding as “the decision to participate in the study may reflect some inherent bias in the characteristics/traits of the participants” [51].

Secondly, underrepresentation (employees who have not signed up to Facebook are not able to participate) and non-response (respondents not starting the survey) are two other risks affecting overall generalisability [52]. Lastly, the process of ‘attrition’ [44], or participant drop out [53], caused a disbalance in the eventual number of participants receiving either the intervention message or control message within the sub-groups.

Another issue that needs to be addressed is the smaller sample size than was originally planned [44]. Due to the deadline for this study, data collection had to be stopped after four weeks and by then only half of the desired number of 280 respondents had been reached. This affected the overall methodological rigour and explanatory power of this study as certain sub-groups were fairly small.

The above two paragraphs discuss limitations regarding the data sample used. A limitation related to the content of this study was the intention–behaviour gap [54]. This paper researched expat employees’ willingness to accept a (temporary) salary reduction (their intention) and not their actual behaviour if and when confronted with a pay cut. Several studies conclude that manipulating intention does not automatically lead to changed behaviour [55,56]. According to Sheeran and Webb “current evidence suggests that intentions get translated into action approximately one-half of the time” [54]. While the intention–behaviour gap is important to identify as a limitation of the study, considering the nature and context of the topic, an experiment exploring willingness to accept offers valuable insights. One can imagine that finding an organisation that approves a field experiment on such a sensitive topic is difficult, which may explain the limited existing research available on this topic.

A last possible limitation to be mindful of is the fact that, for a large percentage of the respondents, English was not their native language. Research shows that understanding and interpreting survey questions in a foreign language can be more challenging, especially if the language is complex [57,58]. While it has to be acknowledged that this may have had an influence on survey-takers, it is important to note that English is a widely accepted working language in Qatar and the language used in the survey was kept relatively ‘simple’ to facilitate ease of interpretation.

### 11.3. Further Research Directions

As this study proves, the topic of employees’ willingness to accept a salary reduction is a topic that can be approached from many different angles. In this study, five of the most promising BCTs were assessed and while no significant changes in overall willingness to accept were identified, the results do provide some interesting directions for further research. First of all, a repetition of the same experiment in a few years’ time could give insights into how the context of the pandemic played a role in results. It would be desirable to conduct the study in a nonpandemic context, as additional clarifications and information were obviously not crucial, as opposed to another context in which they would be.

Furthermore, while the COM-B analysis suggested there was not a substantial difference between expat employees and employees working in the country of their nationality, we did not investigate further differences between nationalities as that would have required a larger sample size (Appendix G does provide the non-generalisable findings on region of provenance from our studies). It would allow for testing of one of the originally proposed explanatory hypotheses: whether regional provenance plays a role in willingness to accept a reduction. This links to Hofstede’s claim that different countries have differing sets of national dimensions [59], raising the question whether certain societies are more inclined than others to accept a temporary pay reduction, especially with regard to differences in the ‘power distance between subordinates and managers’ and ‘individualism vs collectivism in society’ dimensions.

In the same vein, this study established a number of interesting findings when comparing different income situation categories. A more formal, dedicated study into the impact of employees’ financial situation on their willingness to accept could provide additional understanding.

Lastly, taking a broader perspective on this study, including the COM-B analysis, the areas of trust, external opportunities elsewhere, and availability of a financial buffer were not further explored in the context of this study but are likely to play a central role in employees’ decision making and behaviour.

### 11.4. Conclusions

This research aimed to better understand whether the use of multiple BCTs in a CEO’s email announcement increases expat employees’ overall willingness to accept a (temporary) salary reduction. Quantitative analysis shows that while the direction of impact was positive, the compound effect of all five BCTs on overall willingness to accept was not statistically significant. While the smaller than preferred sample size affects generalisability, the results suggest that other factors such as the income level and available financial buffer (as perceived by the employee) and the strength of the employer–employee relationship play a role as well when it comes to accepting a temporary salary reduction.

An important finding of this study with regard to the effect of individual BCTs is that this study confirms that actions of senior management matter when it comes to expat employees’ willingness to accept a pay reduction. The study 1 survey clearly showed that, when asked directly, employees find it very important that their leadership team shares the pain by also undergoing a salary reduction similar to or greater than the employees themselves. Moreover, the study 2 experiment indicated that expat employees are significantly more willing to accept a temporary salary reduction when the message makes clear that senior management will accept a larger reduction compared with the employees participating in the experiment.

In addition, the exploratory component of this study, the analysis of the overall willingness to accept by sub-groups divided by income level (as identified by the participants themselves), provided outcomes that merit further study. Not only does this study suggest that employees on a lower level of income are significantly less likely to accept a temporary salary reduction (regardless of whether they receive the treatment or not), but it also found the unexpected result that those in the ‘Getting By’ category are significantly more willing to accept a temporary pay reduction after receiving the treatment. This effect was not found for the other financial categories.

Another contribution of this study is that it brought a new approach, Behavioural Science, to the Human Resource Management domain. To our knowledge, this is the first application of the BCW method to address HR-related matters and especially to gain a deeper understanding of this specific issue. The more exploratory COM-B analysis phase including a review of the existing literature, employer interviews, and an employee survey gave a thorough introduction to the topic and the many factors playing a role in employee’s decision making. Moreover, the outcomes provided a clear underpinning for the most promising BCTs to use in the experiment. Another pioneering element of this study is its target group, expat employees, and country, Qatar. Often research on HR topics takes place in Western environments and focuses on the ‘local’ population of employees.

The fact that overall willingness to accept a salary reduction did not significantly increase raises the interesting question for future research whether BCTs work equally well when loss aversion plays a role among participants. Possible other explanations for the result relate to the participants’ perceived financial buffer and the influence of the existing employer–employee relationship on their willingness to accept a (temporary) reduction. Moreover, the salience of the pandemic and its consequences could have influenced the effect of the BCTs as, for example, participants were already aware of reasons why their organisation would likely have to take these measures and even the control group would have been sufficiently familiar with the extra explanation given to the treatment group. Future studies could focus on any of the above aspects.

A practical recommendation for leaders in organisations dealing with salary reductions is to be aware of the impact their actions and words can have on employees. Our study suggests that being a role-model and displaying the behaviour one would like to see from their employees in these circumstances increases expat employees’ willingness to accept such a reduction. This can be of critical importance to keep employees motivated when the organisation is in financial distress.

The economic consequences of the pandemic brought to the forefront the practice of temporary salary reductions to weather a financial storm, an issue that had received little attention during the prosperous economic times that preceded the unprecedented event. This study’s overall contribution, therefore, is to help close the knowledge gap through the novel application of the BCW approach to this topic and focussing on the perspective of a distinct group of employees based in a middle eastern country.

## Figures and Tables

**Table 1:** COM-B employees’ survey—results for each component (presented as Minimum, Maximum, Mean, Variance, Standard Deviation).

Rank	Component	Question/Statement	Minimum	Maximum	Mean	Variance	Standard Deviation
1	Opportunity	Senior management would have to accept an equal or larger reduction as well.	1	4	1.23	0.35	0.59
			1	4	1.24	0.35	0.59
2	Capability	I would have to know more about why it is important to accept a salary reduction.	1	5	1.37	0.43	0.65
			1	4	1.35	0.35	0.59
3	Opportunity	There would have to be more people within my organization accepting a salary reduction as well.	1	5	1.63	0.8	0.89
			1	5	1.67	0.95	0.97
4	Motivation (Emotion)	The thought of accepting a reduction in my salary makes me feel anxious.	1	5	1.64	0.9	0.95
			1	5	1.71	1.22	1.1
5	Capability	I would have to be better able to oversee the consequences of a salary reduction for me personally (e.g., the impact of this on my financial or social situation).	1	5	1.72	0.85	0.92
			1	5	1.75	0.94	0.97
6	Opportunity	I would have to be aware of an external event, such a financial crash or the current pandemic, causing damage to the economy and organizations.	1	4	1.77	0.61	0.78
			1	4	1.81	0.71	0.84
7	Trust/ Relationship	I would be more inclined to accept a salary reduction during difficult times in case I aB0 benefited financially when the organization experienced ‘good times’.	1	5	1.84	1.22	1.1
			1	5	1.92	1.38	1.17
8	Motivation	I would have to belief that it would be the right thing to do.	1	5	1.86	1.16	1.08
				5	1.95	1.4	1.18
9	Trust/ Relationship	The relationship (loyalty and trust) I have with the organization I work for affects my willingness to accept a salary reduction.	1	5	1.94	1.51	1.23
			1	5	2.06	1.84	1.36
10	Motivation	For me to accept a salary reduction, I would have to get a positive feeling from helping the organization survive.	1	5	1.94	1.09	1.05
			1	5	1.95	1.11	1.05
11	Opportunity	I would have to have more savings or have other financial buffers.	1	5	2.03	1.05	1.02
			1	5	2.07	1.1	1.05
12	Motivation	I would have to feel that there will be negative effects, such as possible job losses, if I do not accept a salary reduction.	1	5	2.14	1.12	1.06
			1	5	2.27	1.14	1.07
13	Capability	I would have to know more about how to accept a salary reduction.	1	5	2.25	1.47	1.21
				5	2.3	1.39	1.18
14	Opportunity	I would have to have a higher income to start With.	1	5	2.32	1.27	1.13
			1	5	2.32	1.31	1.14
15	Motivation	The thought of accepting a reduction in my fringe benefits makes me feel anxious.	1	5	2.48	1.05	1.02
			1	5	2.31	1.13	1.06
16	Capability	I would have to first overcome the urge to not want to think about a salary reduction.	1	5	2.86	1.73	1.32
			1	5	3.02	1.71	1.31
17	Motivation (Emotion)	If I did not participate in a company-wide salary reduction scheme, I would feel guilty.	1	5	2.94	1.88	1.37
			1	5	2.96	1.87	1.37
18	Opportunity	I would have to have support from family and /or friends in accepting the reductions	1	5	2.98	1.85	1.36
			1	5	3.24	1.77	1.33
19	Motivation	For me to accept a salary reduction, I would have to get a positive feeling from aiding the economic recovery of my country or the country that I live in).	1	5	2.99	1.7	1.3
			1	5	3.06	1.74	1.32
20	Opportunity	There would have to be more people externally (outside my organization) accepting a salary reduction as well.	1	5	3.11	1.72	1.31
			1	5	3.23	1.84	1.36

**Table 2:** COM-B analysis.

COM-B Components	What Needs to Happen for the Target Behaviour to Occur?	Is There a Need for Change? (Based on Literature Review and/or Survey)
Physical capability	Having the physical skills to accept a reduction.	No—No change is needed, as there are no physical barriers to accepting a reduction.
**Psychological capability**	Understanding the reasons for a salary reduction and being able to oversee the impact of this on one’s personal situation.	Yes—The existing literature indicates that an elaborate explanation compared with a shorter one increases understanding among employees and decreases its negative effects. The empirical research among employers and employees also gives great weight to the importance of making a clear case to employees as to why a reduction is the best and possibly only option.
**Physical opportunity**	Having sufficient financial means to absorb the decrease in the employees’ salary.	Yes—Especially for lower incomes or employees who enjoy a lifestyle that uses up all income, as this can be a real barrier to accepting a reduction. At the same time, due to influences such as loss aversion, it is quite likely that even people who could objectively absorb a change in their finances would like to have more reserves or a higher income to start with.
**Social opportunity**	Creation of a social environment that gives clues about the ‘new’ social norm where others also accept a reduction.	Yes—Both the literature and our COM-B research suggest that employees are more willing to accept a reduction when all employees are equally affected (social norm) and senior management leads by example. The creation of a trustful relationship between employer and employee can be a factor in this.
**Reflective motivation**	Holding the belief that it is the right thing to do. Optimism that the decision will have the desired effect. Reflecting on the consequences of not accepting a reduction for both the individual and group.	Yes—Beliefs about it being the right decision to take for the individual and the company are ranked as important by employees.
**Automatic motivation**	Eliciting but also managing the emotional reaction to the request to accept a reduction.	Yes—The survey clearly shows that the thought of a salary reduction makes many employees feel anxious and the literature indicates it can increase feelings of job dissatisfaction.

**Table 3:** Intervention functions selected based on APEASE criteria and pre-defined mode of delivery.

Candidate Intervention Functions	Does Intervention Meet the APEASE Criteria? Affordability, Practicability, Cost-Effectiveness, Acceptability, Side-Effects/Safety, Equity	Can the Intervention Function Be Delivered by the Pre-Defined Mode of Delivery? Communication or Service Provision
**Education**	Yes—increasing knowledge on the reason for the reduction	Yes
**Persuasion**	Yes	Yes
**Incentivisation**	Yes	Yes
Coercion	Not acceptable (although a salary reduction itself could be perceived as punishment)	Yes
Training	Not practicable	Only service provision
Restriction	Not acceptable	No
Environmental Restructuring	Yes—changing the social context	No
**Modelling**	Yes	Yes
**Enablement**	Yes	Only service provision

**Table 4:** Individual BCTs proposed by linking the intervention functions with BCTs best suited for the experiment.

Individual BCTs	Intervention Function(s)	APEASE Criteria Met?	Suitable for Mode of Delivery? (CEO’s Message)
Information about social and environmental consequences	Education, Persuasion	YES	YES—incorporate that many employees worldwide have to accept a salary reduction
Information about consequences	Education Persuasion	YES	YES—on a ‘group’ level (e.g., job losses), not regarding the participant’s personal situation
Demonstration of the behaviour	Modelling	YES	YES
Social support (enablement)	Enablement	YES	YES—the message could refer employees to HR who can help give specific information on the individual’s case

**Table 5:** BCTs and their definition selected for inclusion in the experiment. Source definitions: Michie et al. [5].

	BCT	Definition	Used in This Research in the Following Manner:
BCT 1	**Information/** **Explanation**	From the TDF framework—knowledge domain: information provision [43]	*An extra sentence explaining in more detail the reasons why a salary reduction is necessary.*
BCT 2	**Modelling**	Demonstration of the behaviour: provide an observable sample of the performance of the behaviour directly or indirectly for the person to aspire to or imitate	*A specific reference that senior management will take a larger pay cut (30% as opposed to the 20% asked from participants).*
BCT 3	**Negative Consequences**	Provide information about (health) consequences of performing the behaviour	*An extra sentence indicating that job losses will be inevitable if a temporary salary reduction is not implemented.*
BCT 4	**Empathy**	Not included in Michie et al.’s [5] taxonomy	*The CEO expressing remorse that he/she has to take this decision.*
BCT 5	**Social Support**	Advise on, arrange, or provide social support (e.g., from friends, relatives, colleagues, buddies, or staff)	*More specific HR contact details (telephone number) and offer to provide access to a financial specialist if needed.*

**Table 6:** Descriptive Statistics.

Variable	Descriptive Overview	Randomization Checks		
	Total (N = 140)	Control (N = 78)	Treatment (N = 62)	Difference (*p*-Value)
Gender				χ^2^ 0.53
Male	103 (73.6%)	59	44	
Female	37 (26.4%)	19	18	
Age				χ^2^ 0.41
18–24	6 (4%)	5	1	0.16
25–34	44 (31%)	24	20	0.85
35–44	41 (29%)	20	21	0.28
45–54	38 (27%)	21	17	0.95
54–65	11 (8%)	8	3	0.23
Region				χ^2^ 0.62
Africa	16 (11%)	7	9	0.3
Asia/Pacific	81 (58%)	45	36	0.96
Middle East	20 (14%)	11	9	0.94
Western	23 (16%)	15	8	0.32
Financial Situation				χ^2^ 0.29
Very Comfortable	10 (7%)	5	5	0.7
Comfortable	32 (23%)	22	10	0.09
Getting By	32 (23%)	19	13	0.63
Difficult	33 (24%)	14	19	0.08
Very Difficult	33 (24%)	18	15	0.87
Number of Adult Dependents				χ^2^ 0.41
None	23 (16%)	11	12	0.4
1	21 (15%)	9	12	0.19
2–3	59 (42%)	36	23	0.28
More than 4	37 (26%)	22	15	0.59
Number of Children Dependents				χ^2^ 0.29
None	60 (43%)	33	27	0.88
1	30 (21%)	17	13	0.9
2–3	47 (34%)	28	19	0.51
More than 4	3 (2%)	0	3	0.08

**Table 7:** Comparisons between the conditions based on mean differences, tested with both parametric and non-parametric statistical tests.

Dependent Variable	Control Mean (SD); N = 78	Treatment Mean (SD); N = 62	Difference (P1) [P2] ^ꝴ^
Overall Willingness (1–10)	4.06 (3.01)	4.39 (2.95)	0.33 (0.52) [0.49]
BCT-Specific Willingness (1–7)			
BCT1: Information/Explanation	4.22 (1.66)	4.06 (1.30)	−0.15 (0.55) [0.45]
BCT2: Modelling	3.68 (1.71)	4.32 (1.74)	0.64 ** (0.03) [0.021]
BCT3: Negative Consequences	4.02 (1.71)	4.43 (1.57)	0.41 (0.14) [0.26]
BCT4: Empathy	3.92 (1.58)	4.32 (1.38)	0.4 (0.12) [0.055]
BCT5: Social Support	3.59 (1.52)	4.06 (1.49)	0.47 * (0.06) [0.05]

Note: ** *p* < 0.05, * *p* < 0.1. Difference is computed as ‘Treatment Mean–Control Mean’ ^ꝴ^ P1 corresponds to *p*-values from parametric *t*-test and P2 corresponds to *p*-values from non-parametric Mann–Whitney test.

**Table 8:** Multivariate linear regression analysis for the overall willingness to accept a temporary salary reduction. Primary Analysis: Overall Willingness to Accept a Reduction in Salary.

Primary Outcome Measure	Dependent Variable: Overall Willingness to Accept a Salary Reduction (1–10)							
	Without Controls (N = 140)				With Controls (N = 140)			
Independent Variable	Estimate	SE	*p*-Value	95% CI	Estimate	SE	*p*-Value	95% CI
Treat	0.63	0.47	0.19	−0.32, 1.57	0.47	0.49	0.34	−0.51, 1.45
Financial Situation								
Getting By	−0.65	0.67	0.33	−1.96, 0.67	−0.64	0.65	0.32	−1.94, 0.65
Difficult	−2.35 ***	0.61	0.00	−3.56, −1.14	−2.37 ***	0.65	0.00	−3.65, −1.08
Very Difficult	−2.43 ***	0.7	0.001	−3.83, −1.03	−2.59 ***	0.75	0.001	−4.07, −1.1
Male					−0.93 *	0.53	0.086	−1.99, 0.13
Age								
35–44					1.06	0.64	0.10	−0.21, 2.33
45–54					1.14 *	0.58	0.054	−0.21, 2.3
54–54					−0.79	0.79	0.32	−2.37, 0.78

Note: Financial situation represents a [3 × 1] vector of 3 dummy variables corresponding to the respondents’ financial situation (Getting By, Difficult, Very Difficult), with the remaining one (Very Comfortable/Comfortable) left as a reference category. Robust standard errors are computed. Controls include gender, age, and nationality dummies. Female and age group 18–34 are employed as reference categories. *** *p* < 0.01, * *p* < 0.1.

**Table 9:** Multivariate linear regression analysis for the five Behavioural Change Techniques (BCTs). Secondary Analysis: BCT-specific Willingness to Accept a Reduction in Salary.

Secondary Outcome Measures	BCT-Specific Willingness to Accept a Salary Reduction							
	Without Controls (N = 140)				With Controls (N = 140)			
	Estimate	SE	Unadjusted *p*-Value	FWER q-Value	Estimate	SE	Unadjusted *p*-Value	FWER q-Value
Willingness to Accept (1–7)								
BCT1: Information/Explanation	−0.065	0.25	0.79	0.79	−0.13	0.26	0.617	0.62
BCT2: Modelling	**0.75**	**0.29**	0.01 **	0.06 *	0.68	0.3	0.025 **	0.12
BCT3: Negative Consequences	0.48	0.28	0.087 *	0.26	0.39	0.28	0.17	0.47
BCT4: Empathy	0.41	0.25	0.107	0.26	0.37	0.26	0.156	0.47
BCT5: Social Support	0.49	0.26	0.065 *	0.26	0.5	0.27	0.064 *	0.26

Note: ** *p* < 0.05, * *p* < 0.1. All regressions control for the stratification variable (financial situation) defined in terms of 4 categories (Very Comfortable/Comfortable, Getting By, Difficult, and Very Difficult). Unadjusted *p*-values are *p*-values computed based on the t-distribution. FWER q-values adjust for multiple testing and are computed using Holm–Bonferroni method. Controls include gender and age.

**Table 10:** Heterogeneous Analysis: Overall Willingness to Accept a Reduction in Salary.

Independent Variable	Dependent Variable: Overall Willingness to Accept a Salary Reduction (Coefficient on the Treat Variable)	
	Estimate (95% CI)	*p*-Value
**Financial Situation ^1^**		
Very Comfortable/Comfortable	−0.75 (−2.58, 1.07)	[0.417]
Getting By	2.75 *** (1.14, 4.36)	[0.001] {0.012}
Difficult	1.1 (−0.5, 2.71)	[0.176]
Very Difficult	−0.69 (−2.96, 1.57)	[0.55]
*Differential Effect (2nd–1st)*	3.5 *** (1.04, 5.96)	[0.006]
*Differential Effect (2nd–4th)*	3.44 ** (−0.66, 6.22)	[0.016]
*Differential Effect (3rd–1st)*	1.86 (−0.58, 4.3)	[0.13]
*Differential Effect (3rd–4th)*	1.8 (−1.0, 4.6)	[0.2]
**Age**		
18–34	1.53 * (0.24, 3.31)	[0.09]
35–44	0.77 (−1.09, 2.64)	[0.41]
45–54	−1.12 (−2.55, 0.31)	[0.12]
55–64	0.17 (−2.21, 2.56)	[0.88]
*Differential Effect (3rd–1st)*	−2.66 ** (−4.94, −0.37)	[0.02]
*Differential Effect (3rd-2nd)*	−1.89 (−4.27, 0.47)	[0.11]
**Gender**		
Male	0.2 (−0.89, 1.31)	[0.71]
Female	1.06 (−0.77, 2.88)	[0.25]
*Differential Effect (Female–Male)*	0.85 (−1.28, 2.99)	[0.43]
**Nationality**		
Asian	1.24 * (−0.08, 2.57)	[0.066]
Middle East/Africa	−0.52 (−2.23, 1.19)	[0.55]
Western	−0.27 (−2.47, 1.9)	[0.8]
*Differential Effect (2nd–1st)*	−1.76 (−3.87, 0.35)	[0.10]

Note: Confidence intervals are reported in parentheses, unadjusted *p*-values in brackets [], and adjusted q-values using the Holm–Bonferroni method in brackets {}. *** *p* < 0.01, ** *p* < 0.05, * *p* < 0.1. ^1^ Stratification Variable.

**Figure A1:**
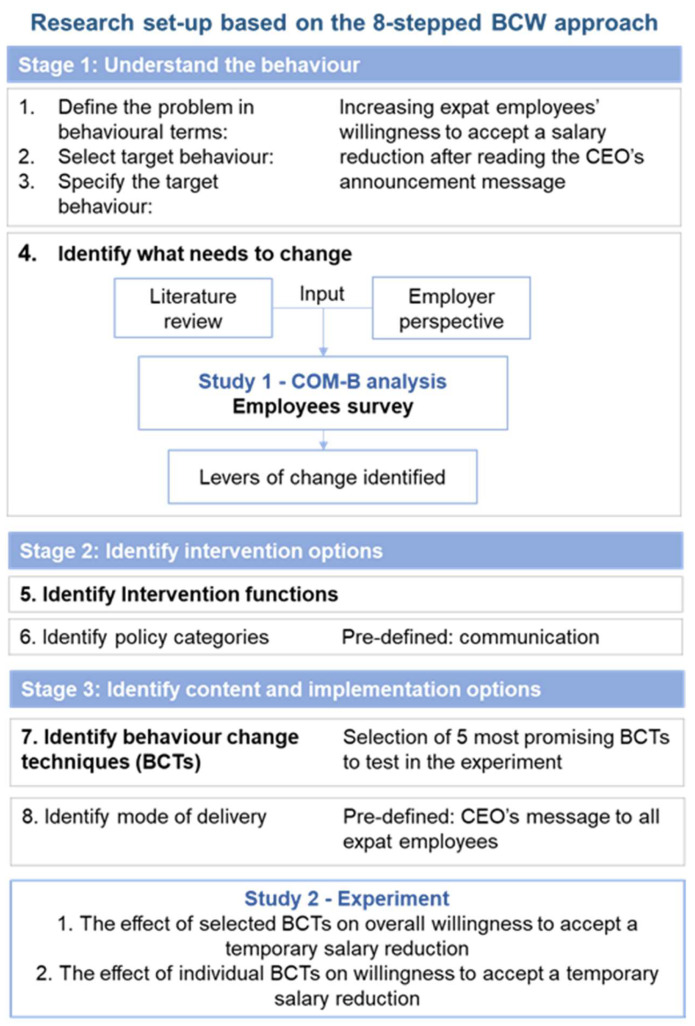
Research Set-up.

## Data Availability

The datasets generated during and/or analysed during the current study are available from the corresponding author on reasonable request.

## Appendix A

**Table A1:** Heterogeneous Analysis: BCT-specific Willingness to Accept a Reduction in Salary (same as Table 10 but with additional controls).

Independent Variable	Dependent Variable: Willingness to Accept Salary Reduction (Coefficient on the Treat Variable)				
	BCT1 Explanation	BCT2 Modelling	BCT3 Negative Consequence	BCT4 Empathy	BCT5 Social Support
**Panel A: Financial Situation ^1^**					
Very Comfortable/Comfortable	−0.43 (0.32)	**1.14 ** (0.018)**	0.49 (0.27)	0.14 (0.73)	0.46 (0.28)
Getting By	0.62 (0.16)	**1.18 ** (0.049)**	**1.17 ** (0.027)**	**1.1 *** (0.008)**	**0.89 * (0.08)**
Difficult	−0.66 (0.14)	0.22 (0.71)	0.18 (0.75)	0.04 (0.93)	−0.26 (0.65)
Very Difficult	0.15 (0.82)	0.21 (0.77)	−0.11 (0.88)	0.39 (0.58)	0.99 (0.12)
**Panel B: Age**					
18–34	0.19 (0.7)	0.84 (0.106)	0.57 (0.27)	0.7 (0.17)	0.46 (0.38)
35–44	−0.14 (0.74)	**1.07 ** (0.035)**	0.79 (0.108)	0.2 (0.64)	**1.01 ** (0.023)**
45–54	−0.59 (0.17)	0.18 (0.76)	−0.18 (0.72)	0.07 (0.87)	−0.05 (0.9)
55–64	0.43 (0.54)	0.45 (0.54)	0.54 (0.49)	**0.94 * (0.058)**	0.97 (0.18)
**Panel C: Gender**					
Male	−0.077 (0.81)	**0.53 (0.16)**	0.37 (0.28)	0.33 (0.31)	0.3 (0.36)
Female	−0.27 (0.48)	**1.1 ** (0.025)**	0.42 (0.36)	0.49 (0.23)	**1.02 ** (0.015)**
**Panel D: Nationality**					
Asian	0.42 (0.25)	**0.89 ** (0.025)**	**0.73 * (0.07)**	**0.71 * (0.054)**	**0.72 ** (0.047)**
Middle East/Africa	**−1.15 *** (0.004)**	0.05 (0.93)	**−0.39 (0.47)**	**0.16 (0.76)**	**0.31 (0.59)**
Western	−0.44 (0.37)	**1.12 ** (0.049)**	0.63 (0.21)	−0.11 (0.8)	0.2 (0.7)

Note: Unadjusted *p*-values are reported in parentheses. *** *p* < 0.01, ** *p* < 0.05, * *p* < 0.1. All regressions control for income and age. ^1^ Stratification Variable.

## Appendix B. BCW Definitions—Intervention Functions and Selected BCTs

**Table A2:** The nine intervention functions and their definition. Source definitions and examples: Michie et al. [5].

Intervention Function	Definition	Example of Intervention Function
**Education**	Increasing knowledge or understanding	*Providing information to promote healthy eating*
**Persuasion**	Using communication to induce positive or negative feelings or stimulate action	*Using imagery to motivate increases in physical activity*
**Incentivisation**	Creating an expectation of reward	*Using prize draws to induce attempts to stops smoking*
**Coercion**	Creating an expectation of punishment or cost	*Raising the financial cost to reduce excessive alcohol consumption*
**Training**	Imparting skills	*Advanced driver training to increase safe driving*
**Restriction**	Using rules to reduce the opportunity to engage in the target behaviour (or to increase the target behaviour by reducing the opportunity to engage in competing behaviours)	*Prohibiting sales of solvents to people under 18 to reduce use for intoxication*
**Environmental restructuring**	Changing the physical or social context	*Providing on-screen prompts for GPs to ask about smoking behaviour*
**Modelling**	Providing an example for people to aspire to or imitate	*Using TV drama scenes involving safe-sex practices to increase condom use*
**Enablement**	Increasing means/reducing barriers to increase capability (beyond education and training) or opportunity (beyond environmental restructuring)	*Behavioural support for smoking cessation, medication for cognitive deficits, surgery to reduce obesity.*

**Table A3:** BCTs and their definition selected for inclusion in the experiment. Source definitions: Michie et al. [5].

	BCT	Definition	Used in This Research in the Following Manner:
BCT 1	**Information/** **Explanation**	From the TDF framework—knowledge domain: information provision (French et al. [43])	*An extra sentence explaining in more detail the reasons why a salary reduction is necessary.*
BCT 2	**Modelling**	Demonstration of the behaviour: provide an observable sample of the performance of the behaviour directly or indirectly for the person to aspire to or imitate	*A specific reference that senior management will take a larger pay cut (30% as opposed to the 20% asked from participants).*
BCT 3	**Negative Consequences**	Provide information about (health) consequences of performing the behaviour	*An extra sentence indicating that job losses will be inevitable if a temporary salary reduction is not implemented.*
BCT 4	**Empathy**	Not included in Michie et al.’s [5] taxonomy	*The CEO expressing regret/remorse that he has to take this decision.*
BCT 5	**Social Support**	Advise on, arrange, or provide social support (e.g., from friends, relatives, colleagues, buddies, or staff)	*More specific HR contact details (telephone number) and offer to provide access to a financial specialist if needed.*

## Appendix C. Demographic Details (Study 1)

Demographics of all 155 participants:

Expat-only demographics (84 participants):

## Appendix D. COM-B Survey Questions (Study 1)

**Component**	**Question**
Psychological capability	I would have to know more about why it is important to accept a salary reduction.
	I would have to know more about how to accept a salary reduction.
	I would have to be better able to oversee the consequences of a salary reduction for me personally (e.g., better understand the impact of this on my financial or social situation).
	I would have to first overcome the urge to not want to think about a salary reduction.
Physical opportunity	I would have to have more savings or have other financial buffers.
	I would have to have a higher income to start with.
Social Opportunity	There would have to be more people within my organisation accepting a salary reduction as well.
	Senior management would have to accept an equal or larger reduction as well.
	There would have to be more people externally (outside my organisation) accepting a salary reduction as well.
	I would have to be aware of an external event, such a financial crash or the current pandemic, causing damage to the economy and organisations.
	I would have to have support from others such as family and/or friends in accepting the reduction
Reflective Motivation	For me to accept a salary reduction, I would have to get a positive feeling from helping the organisation survive.
	For me to accept a salary reduction, I would have to get a positive feeling from aiding the economic recovery of my country (or the country that I live in).
	I would have to feel that there will be negative effects, such as possible job losses, if I do not accept a salary reduction.
	I would have to belief that it would be the right thing to do.

**Domain/** **Factor**	**Statement**
Emotion (TDF) Linked to COM-B automatic motivation component	The thought of accepting a reduction in my fringe benefits makes me feel anxious.
	The thought of accepting a reduction in my salary makes me feel anxious.
	If I did not participate in a company-wide salary reduction scheme, I would feel guilty.
Trust/ Relationship	I would be more inclined to accept a salary reduction during difficult times in case I also benefited financially when the organisation experienced ‘good times’.
Trust/ Relationship	The relationship (loyalty and trust) I have with the organisation I work for affects my willingness to accept a salary reduction.

## Appendix E. Individual BCTs Considered to Best Serve the Defined Intervention Functions

**Table A4:** 21 BCTs considered to best serve the defined intervention functions. Source definitions: Michie et al. [5].

Individual BCTs	Definition	Intervention Function(s)	APEASE Criteria Met?	Suitable for Mode of Delivery? (CEO’s Message)
**Information about social and environmental consequences**	Provide information about social and environmental consequences of performing the behaviour	Education, Persuasion	Yes	Yes
**Information about (health) consequences**	Provide information about (health) consequences of performing the behaviour	Education Persuasion	Yes	Yes—on a ‘group’ level (e.g., job losses), not regarding the personal situation
**Feedback on behaviour**	Monitor and provide information or evaluative feedback on performance of the behaviour	Education, Persuasion, Incentivisation	Not relevant in this context	-
**Feedback on outcome(s) of behaviour**	Monitor and provide feedback on the outcome of performance of the behaviour	Education, Persuasion, Incentivisation	Not relevant in this context	-
**Prompts/cues**	Introduce or define environmental or social stimulus with the purpose of prompting or cueing the behaviour. The prompt would normally occur at the time or place of performance	Education	Yes/No—prompts regarding the effect of the pandemic on the economy are given daily	Yes/No—Seeing news activity around COVID-19 as ‘prompt’ relevant but cannot be especially introduced in this message. Not a BCT to test
**Credible source**	Present verbal or visual communication from a credible source in favour of or against the behaviour	Persuasion	Yes	No—participants are asked in the experiment to imagine the message comes from their own CEO. Not a BCT to test
**Social comparison**	Draw attention to others’ performance to allow comparison with the person’s own performance	Persuasion	Yes	No—the salary reduction in our experiment is not voluntary. Moreover, the message is an announcement of a reduction applicable to all
**Monitoring of behaviour by others without evidence of feedback**	Observe or record behaviour with the person’s knowledge as part of a behaviour change strategy	Incentivisation	Not relevant in this context	-
**Monitoring outcome of behaviour by others without evidence of feedback**	Observe or record outcomes of behaviour with the person’s knowledge as part of a behaviour change strategy	Incentivisation	Not relevant in this context	-
**Self-monitoring of behaviour**	Establish a method for the person to monitor and record their behaviour(s) as part of a behaviour change strategy	Incentivisation	Not relevant in this context	-
**Demonstration of the behaviour**	Provide an observable sample of the performance of the behaviour, directly in person or indirectly, e.g., via film, pictures for the person to aspire to or imitate.	Modelling	Yes	Yes
**Social support (unspecified)**	Advise on, arrange or provide social support for performance of the behaviour	Enablement	Yes	Yes—the message could refer employees to HR who can help give specific information on the individual’s case
**Social support (practical)**	Advise on, arrange, or provide practical help for performance of the behaviour	Enablement	Not relevant in this context	-
**Goal setting (behaviour)**	Set or agree a goal defined in terms of the behaviour to be achieved	Enablement	Not relevant in this context	-
**Goal setting (outcome)**	Set or agree a goal defined in terms of a positive outcome of wanted behaviour	Enablement	Not relevant in this context	-
**Adding objects to the environment**	Add objects to the environment in order to facilitate performance of the behaviour	Enablement	Not relevant in this context	-
**Problem solving**	Analyse, or prompt the person to analyse, factors influencing the behaviour and generate or select strategies that include overcoming barriers and/or increasing facilitators	Enablement	Yes	No—group message does not lend itself to individual analysis of factors influencing the behaviour
**Action planning**	Prompt detailed planning of performance of the behaviour	Enablement	Not relevant in this context	-
**Restructuring of the physical environment**	Change, or advise to change, the physical environment in order to facilitate performance of the wanted behaviour or create barriers to the unwanted behaviour	Enablement	Not relevant in this context	-
**Review behaviour goal(s)**	Review behaviour goal(s) jointly with the person and consider modifying goal(s) or behaviour change strategy in light of achievement	Enablement	Not relevant in this context	-
**Review outcome goal(s)**	Review outcome goal(s) jointly with the person and consider modifying goal(s) in light of achievement	Enablement	Not relevant in this context	-

## Appendix G. Experiment—Full Text CEO’s Message Including BCTs (Study 2)

*Imagine your current organisation has gotten into economically difficult times. In our example we use the current COVID-19 pandemic. If your own organisation is currently not impacted, picture a scenario where another kind of external event is affecting not only your specific organisation but the entire sector that it is active in.*

*You are going to read six short parts of an e-mail message from your CEO. In this message, your CEO will announce a 20% salary reduction for at least six months until there are clear signs of recovery within the sector you work in.*

*For research purposes, we have placed the different parts of this e-mail in a random order. It could therefore be that you do not see the email in a logical order.*

*Please indicate for each part how reading that part of the text has influenced your willingness to accept a salary reduction.*

*[Randomised—BCT sentences included in the intervention message underlined]*

I am truly sorry to have to ask you to give even more during a situation where many of you have already worked extremely hard to keep the company afloat, often while working from home under less than ideal circumstances. There may be questions as to whether these actions will be enough. Although the market conditions are difficult and recovery may not be as rapid as we wish, we are confident that our updated business plan is sufficiently robust to see us through the worst part of the downturn.
*Please indicate how this paragraph has influenced your willingness to accept a (temporary) salary reduction.*

The current conditions and organizational review have forced me to make a difficult but necessary decision. Starting next month, salaries will be reduced by 20% for at least six months until there are clear signs of recovery. Bonuses will not be paid over the current year and budgets for courses will be withdrawn until further notice. Health insurance coverage and allowances such as those for housing will remain unchanged. We believe senior management should lead by example and shoulder their part of the burden. Therefore, the salaries for the executive team and myself will be reduced by 30%, and we will also forego performance bonuses.
*Please indicate how this paragraph has influenced your willingness to accept a (temporary) salary reduction.*

I can imagine some of you will have further questions regarding your personal financial situation as a result of this message. Please contact your line manager or HR to discuss any further queries you may have. A specific HR line with expertise to answer your questions has been set up and can be contacted between 8am to 8pm on weekdays (4425 5188, HR@organisation.com). They will also be able to refer you to a financial specialist if your financial situation requires this.
*Please indicate how this paragraph has influenced your willingness to accept a (temporary) salary reduction.*

This was an extremely tough decision. However, we considered several other alternatives, and the choice we made is the best option at this time. The immediate cash saving achieved by this measure is necessary to sustain our organisation’s short-term financial health. The sad reality is that if we do not take this measure, forced job losses will be inescapable.
*Please indicate how this paragraph has influenced your willingness to accept a (temporary) salary reduction.*

Dear colleagues, We continue to find ourselves living through extraordinary times as the COVID-19 pandemic keeps on causing health impacts and general disruption. Like so many other sectors of the economy, this crisis has hit our industry hard and it has not left our organisation untouched either. Due to a significant proportion of clients cancelling or postponing their orders, our revenue this year is forecast to be just half of what we earned in 2019. As a result we need to focus on our cash flow the coming period to ensure the company can continue to operate. We anticipate that this situation will last for several more months until confidence is restored. For the past weeks, multiple groups in our organisation have been working tirelessly to give shape to an updated business plan that will implement several measures to achieve cost reduction and efficiency improvement. Further details on the new plan will be shared with you in the coming week. However, via this message I want to announce one specific measure that will impact all of us.
*Please indicate how this paragraph has influenced your willingness to accept a (temporary) salary reduction.*

Take care of yourself and each other. Regards, Peter

*We now would like to ask you two last questions about the message. Take a moment to read the text in logical order below:*

Dear colleagues,

We continue to find ourselves living through extraordinary times as the COVID-19 pandemic keeps on causing health impacts and general disruption. Like so many other sectors of the economy, this crisis has hit our industry hard and it has not left our organisation untouched either. Due to a significant proportion of clients cancelling or postponing their orders, our revenue this year is forecast to be just half of what we earned in 2019. As a result we need to focus on our cash flow the coming period to ensure the company can continue to operate. We anticipate that this situation will last for several more months until confidence is restored. For the past weeks, multiple groups in our organisation have been working tirelessly to give shape to an updated business plan that will implement several measures to achieve cost reduction and efficiency improvement. Further details on the new plan will be shared with you in the coming week. However, via this message I want to announce one specific measure that will impact all of us.

The current conditions and organisational review have forced me to make a difficult but necessary decision. Starting next month, salaries will be reduced by 20% for at least six months until there are clear signs of recovery. Bonuses will not be paid over the current year and budgets for courses will be withdrawn until further notice. Health insurance coverage and allowances such as those for housing will remain unchanged. We believe senior management should lead by example and shoulder their part of the burden. Therefore, the salaries for the executive team and myself will be reduced by 30%, and we will also forego performance bonuses.

This was an extremely tough decision. However, we considered several other alternatives, and the choice we made is the best option at this time. The immediate cash saving achieved by this measure is necessary to sustain our organisation’s short-term financial health. The sad reality is that if we do not take this measure, forced job losses will be inescapable.

I am truly sorry to have to ask you to give even more during a situation where many of you have already worked extremely hard to keep the company afloat, often while working from home under less than ideal circumstances. There may be questions as to whether these actions will be enough. Although the market conditions are difficult and recovery may not be as rapid as we wish, we are confident that our updated business plan is sufficiently robust to see us through the worst part of the downturn.

I can imagine some of you will have further questions regarding your personal financial situation as a result of this message. Please contact your line manager or HR to discuss any further queries you may have. A specific HR line with expertise to answer your questions has been set up and can be contacted between 8am to 8pm on weekdays (4425 5188, HR@organisation.com). They will also be able to refer you to a financial specialist if your financial situation requires this.

Take care of yourself and each other.

Regards,

Peter

*After reading this message, please rate on a scale from 1 to 10 how likely it is that you would accept a salary reduction.*

*SLIDER 1-10*

*Please let us know why you have given the above rating?*

*TEXT BOX*

*END OF SURVEY MESSAGE*
